# Patient-reported toxicity symptoms during tyrosine kinase inhibitor treatment in chronic myeloid leukemia: a systematic review and meta-analysis

**DOI:** 10.1007/s00520-025-09451-4

**Published:** 2025-05-03

**Authors:** Yolba Smit, Pien Scheuter, Myrthe P. M. Lange, Jeroen J. W. M. Janssen, Eduardus F. M. Posthuma, Charlotte L. Bekker, Rosella P. M. G. Hermens, Nicole M. A. Blijlevens

**Affiliations:** 1 Department of Hematology, Radboud university medical center, Route 476, P.O. Box 9101, 6500 HB Nijmegen, The Netherlands; 2 Department of Internal Medicine, Reinier de Graaf Hospital, Delft, The Netherlands; 3 Department of Pharmacy, Radboud university medical center, Nijmegen, The Netherlands; 4 Department of IQ Health, Radboud university medical center, Nijmegen, The Netherlands

**Keywords:** Chronic myeloid leukemia, CML, Patient-reported outcome measure, PROM, Toxicity, Tyrosine kinase inhibitor, TKI, Meta-analysis

## Abstract

**Purpose:**

One in five chronic myeloid leukemia (CML) patients experiences such intolerability that they switch tyrosine kinase inhibitor (TKI) treatment within 3 years. Information on tolerability is needed to guide shared decision-making. However, an overview of symptoms patients experience per TKI is lacking, and physician-graded toxicity underestimates patients’ experiences.

**Methods:**

We systematically searched PubMed and Embase from inception to February 2025 and conducted a meta-analysis on the prevalence of patient-reported symptoms in CML per TKI. This study follows the Preferred Reporting Items for Systematic Reviews (PRISMA) guideline for systematic reviews.

**Results:**

We included 11 studies with 2987 patients, reporting on 47 different symptoms of any severity. The low-grade patient-reported symptom burden was high. No data were available on asciminib and ponatinib, and minimal data were available for bosutinib. In indirect, unadjusted comparisons, 13 out of 47 symptoms (of any severity) showed significant differences in prevalence between common TKI types.

**Conclusion:**

Our findings provide essential information to guide treatment decisions in cases of intolerability. However, there is a clear need for further research with standardized instruments, especially in second and third generation TKI types, including direct comparisons and comparisons adjusted for covariates.

**Supplementary Information:**

The online version contains supplementary material available at 10.1007/s00520-025-09451-4.

## Introduction

For two decades, patients with chronic myeloid leukemia (CML) have been effectively treated with tyrosine kinase inhibitors (TKIs). Currently, six types of TKIs are available in most countries (imatinib, dasatinib, nilotinib, bosutinib, ponatinib, and asciminib) [69]. As patients reach a near-normal life expectancy when they attain an optimal response [9, 58], quality of life during the often lifelong TKI treatment has become increasingly important. The symptom burden during TKI therapy, as reported by patients, has been shown to strongly influence quality of life, treatment adherence, and consequently, treatment response [11, 23, 40]. However, current CML guideline recommendations are primarily based on clinical trials in which survival or disease control is the primary outcome. In these trials, toxicity is primarily evaluated using the Common Terminology Criteria for Adverse Events (CTCAE) [10], graded by physicians. The focus is on clinically relevant toxicities that require medical intervention, such as neutropenia and thrombocytopenia, as well as complications like cardiovascular events [33]. Patient-reported symptoms, such as fatigue and skin problems, are often underreported, as they are deemed less relevant for hematooncological management and thus easily overlooked by physicians. Additionally, symptoms experienced by CML patients during therapy are underestimated by physicians, both in severity and prevalence [21]. However, intolerance leads to switching TKI treatment in one in five patients within the first 3 years treatment initiation [25]. In view of lifelong treatment, needed for most CML patients, efforts to optimize quality of life should be urgently addressed. As part of this, an overview of patient-reported symptoms per TKI is needed, as well as the differences in symptom burden between different TKI types, to adequately support patients, maximizing effectiveness while minimizing symptom burden and informing shared decision-making for treatment choices. Although many reviews, primarily narrative, summarize adverse effects, no quantitative summary of patients’ experiences exists. We therefore systematically reviewed and meta-analyzed the prevalence of toxicity-symptoms during each type of TKI treatment, as reported by CML patients.

## Materials and methods

### Data sources and search strategy

We searched PubMed and Embase/Ovid in English (inception—January 2022, updated in July 2023 and February 2025) with MeSH-terms or similar, plus free text terms—including synonyms and brand names—for CML and the six current TKI types. The full search strategy is given Supplementary File 1. This study is reported according to the Preferred Reporting Items for Systematic Reviews (PRISMA) format [51].

### Eligibility criteria

Articles had to be on chronic phase CML patients ≥ 18 years, treated with imatinib, nilotinib, dasatinib, bosutinib, ponatinib, or asciminib and report the prevalence (proportion) of patients who experienced a symptom separately for each TKI, and for each symptom.

### Study selection

Two authors (YS and either PS or ML) independently selected studies, first on title and abstract, and subsequently full text. Prospective studies that mentioned symptoms or adverse effects in their abstract were always screened full text to determine whether these included patient-reported symptoms. Full-text selections were compared between authors and differences were discussed until consensus. Reference lists of included articles and systematic reviews were checked.

### Data extraction

Data were extracted or calculated by a single researcher (PS or ML) and included the proportion of patients who reported a symptom of any severity (prevalence). If needed, proportions were calculated or extracted visually from figures. For the analysis of moderate-severe symptoms, the proportion of patients who scored one of the top two response options on a four-point scale was extracted. If > 1 proportion was available over time, 12 months was taken, as this was the last measuring point available for all included articles. Symptoms that were described in various ways in different articles were brought together under a general term (see Supplementary File 1). When available, general symptoms (e.g., pain) were described in more detail (e.g., musculoskeletal pain, abdominal pain). In addition, study type, type of questionnaire(s) used, population size, treatment characteristics, median age, gender, comorbidity, and comedication were extracted.

### Critical appraisal

Two researchers independently assessed study quality (PS and ML or YS), using the adapted version of the Newcastle Ottawa Scale [48], resolving differences of opinion through discussion. There is no ideal quality assessment tool designed specifically for prevalence studies [28]; the Newcastle Ottawa Scale has been described as the best option [45] with an adapted version available for prevalence studies [32, 48]. It assesses sampling, sample size, response rate, measurement tool, and outcome assessment. We considered the assessment of confounding factors and statistical tests as not applicable to our data, and a score < 5 as low-quality.

### Statistical analysis

We performed meta-analyses on the prevalence of symptoms per TKI with a random effects model using Stata 17. Zero prevalence was imputed as 0.5 to be included in meta-analysis. The standard error of prevalences was calculated using population size and prevalence. Statistical heterogeneity was assessed using the *I*-squared (*I*^2^) estimate, and considered high (> 50%), moderate (25–50%), or low (< 25%) [35]. We evaluated between-group differences in prevalence, also in indirect comparisons between studies. To limit findings due to chance, because of the high number of comparisons, we imposed *p* < 0.01 instead of *p* < 0.05 as a significancy limit for between-group differences.

We performed sensitivity analysis for the three most common symptoms of each TKI, excluding low-quality studies. For nilotinib, sensitivity analysis was also performed on anxiety and depression and pain as these were the only symptoms one of the low-quality studies described. If significant (*p* < 0.05), low-quality studies would be excluded from meta-analysis.

We explored heterogeneity in a random effects meta-regression analysis, for symptoms described by ≥ 5 articles, testing separately for multiple covariates related to population characteristics (median age (if needed mean age), male proportion, median treatment duration, comorbidity prevalence, comedication prevalence). If ≥ 10 studies with a selected covariate were available, we would perform a multivariate meta-regression [7].

## Results

### Search results and study selection

**Fig. 1:**
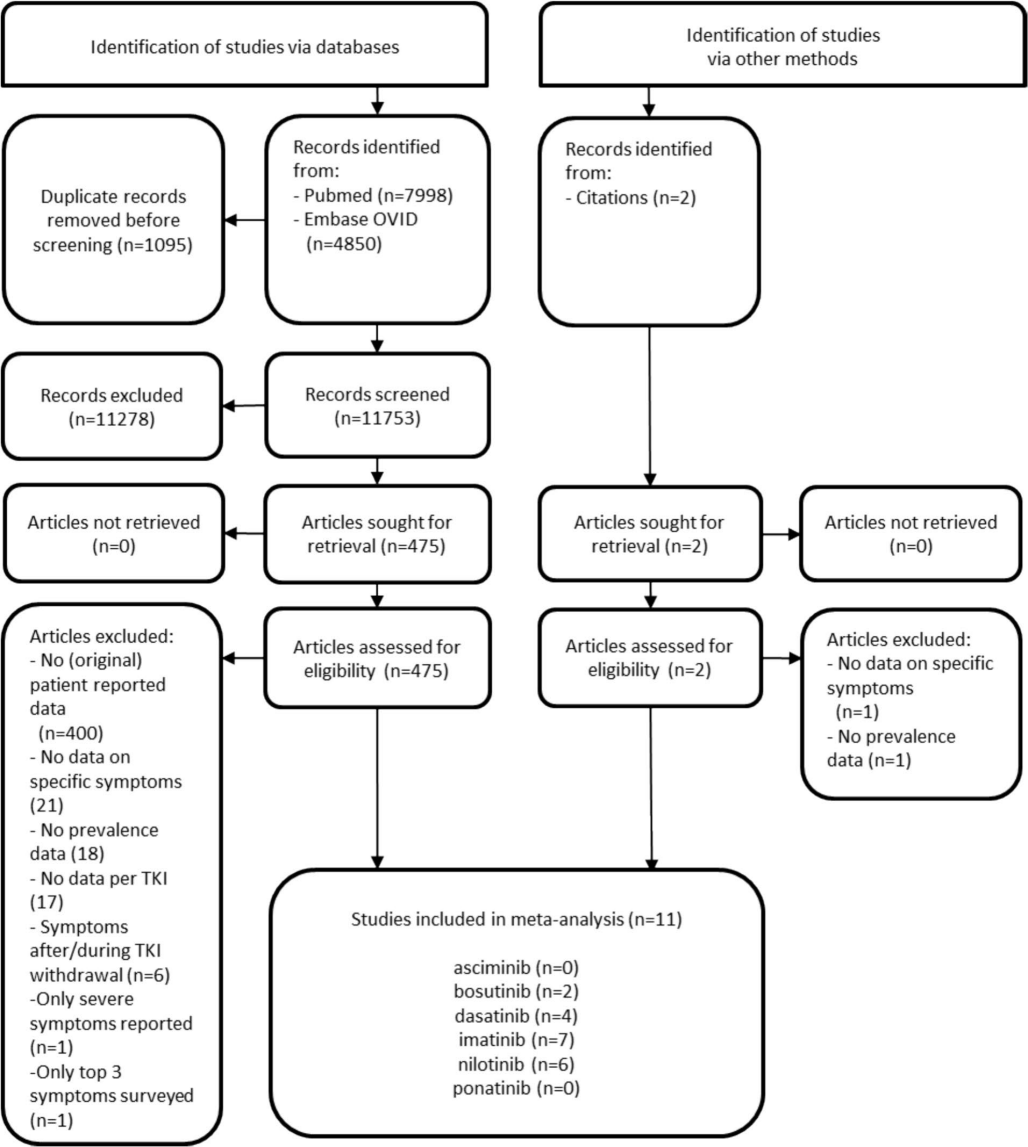
PRISMA flow diagram of study selection

We selected 11 studies with 2987 patients: seven on imatinib [8, 14, 16, 22, 42, 43, 70] (1795 patients), four on dasatinib [8, 22, 43, 70] (233 patients), six on nilotinib [6, 8, 19, 34, 43, 70] (509 patients), two on bosutinib [14, 41] (450 patients), and zero on asciminib or ponatinib (Fig. 1). Sixty-six studies were ineligible because they did not report (original) patient data; no data on specific symptoms were reported; prevalence data was not provided, or not provided per symptom/per TKI, or only gathered for the top three symptoms; symptoms after TKI withdrawal were reported on; or only severe (and not moderate-severe) symptoms were reported on.

### Study characteristics

Four out of eleven included articles were published in the past five years (Table 1). The questionnaires that were used were the European Organization for Research and Treatment for Cancer (EORTC) Quality of Life Questionnaire C30, CML24 (EORTC QLQ-C30, EORTC QLQ-CML24), the EuroQol 5D (EQ- 5D), the functional assessment of cancer therapy leukemia (FACT-Leu), the MD Anderson Symptom Inventory for chronic myeloid leukemia (MDASI-CML), the Patient Health Questionnaire- 9 (PHQ- 9), and generic questionnaires. Population-sizes ranged from nine to 859 per TKI across studies. Treatment duration differed greatly: some studies included patients starting TKI treatment, while maximum median treatment duration was 5 years. In most studies which reported on the line of therapy, at least half of the population had been treated with a different TKI previously. Median age ranged from 40 to 63 years, and the percentage of male patients ranged from 46 to 63%. Comorbidities ranged from 26 to 60%, reported by five articles. Prevalence of comedication was described by three articles and ranged from 15 to 73%.

**Table 1:** Characteristics of included studies

Article	Study type (setting)	Questionnaires (eligibility)	Patients included	TKI type (n of patients)	TKI treatment duration reported by studies	Line of TKI therapy	Starting dose	Age (median)	Male (%)	Comorbidity (%)	Comedication (%)
Boons 2020 [6]	Observational (six Dutch hospitals)	Generic (patients on nilotinib)	68	nilotinib (68)	43% started treatment 57% median 42 m of treatment at study start Symptoms reported at 3, 6, and 12 m	51% 1 st line 49% 2nd/3rd line	72% 2 × 300 mg/d 7% 2 × 150 mg/d 13% 2 × 400 mg/d 3% 300 mg/d 4% 400 mg/d	Mean: 58	51	56	73
Bostan 2020 [8]	Cross-sectional (university hospital, Turkey)	EORTC QLQ-CML24 MDASI-CML (on TKI treatment)	121	Dasatinib (30) Imatinib (61) Nilotinib (30)	Median 31 m	50.4% 1 st line 49.6% 2nd line	Not reported Dose reductions in 8% of patients	53	46	91% HCT-CI < 3	Not reported
Cortes 2019 [14]	RCT (multicenter, international)	EQ- 5D FACT-Leu (ECOG performance status 0 or 1)	385	Bosutinib (194) Imatinib (191)	Treatment started for study Symptoms reported at 12 m	100% 1 st line	Bosutinib 400 mg/d Imatinib 400 mg/d	53	57	Not reported	Not reported
Efficace 2011 [16]	Cross-sectional (26 Italian centers)	Generic (imatinib as 1 st-line ≥ 3 years, in complete cytogenetic response)	422	Imatinib (422)	Median 5 y	Not reported	91% 400 mg/d 4% < 400 mg/d 5% > 400 mg/d Dose changes in 39% of patients	57	59	36.3% ≥ 1 at diagnosis	Not reported
Efficace 2020 [22]	Cross-sectional (38 German and Italian centers)	EORTC QLQ-CML24 (dasatinib or imatinib as 1 st-line < 3 years, in complete cytogenetic response)	188	Dasatinib (94) Imatinib (94)	Median 17 m	22.4% previous treatment	Dasatinib 85% 100 mg/d Imatinib 82% 400 mg/d	63	54	58	59
Huguet 2019 [34]	Observational (multicenter, France)	EQ- 5D- 3L (recently diagnosed, nilotinib as 1 st-line)	98	Nilotinib (98)	Treatment started for study Symptoms reported at 24 m	100% 1 st line	98% 600 mg/day	54	53	Not reported	Not reported
Kantarjian 2018 [41]	RCT (multicenter, international)	EQ- 5D FACT-Leu (resistance/intolerance to imatinib, ECOG 0 or 1)	256	bosutinib (256)	Treatment started for study Symptoms reported at 36, 96, 192, and 360 w	45.7% 2nd line 37.7% 3rd line 16.1% 4 th line 0.5% 5 th line	500 mg/d	Mean: 52	50	Not reported	Not reported
Kapoor 2015 [42]	Cross-sectional (single center, India)	PHQ- 9 (imatinib ≥ 3 months, < 80 years)	100	imatinib (100)	Median 30 m	Not reported	81% 400 mg/d 13% 600 mg/d 6% 800 mg/d	40	63	Not reported	15
Kekale 2015 [43]	Cross-sectional (eight secondary and tertiary care hospitals in Finland)	Generic (TKI treatment ≥ 6 months)	86	Dasatinib (9) Imatinib (68) Nilotinib (9)	Not reported	54.7% 1 st line 29.1% 2nd line 15.1% 3rd line 1.2% 4 th line	Not reported	59	52	Median 1 per patient	Median 2 per patient
Nguyen 2022 [49]	Cross-sectional (two Vietnamese centers)	EORTC QLQ-C30 (resistant/intolerant to imatinib, on nilotinib ≥ 3 months)	121	Nilotinib (121)	Mean 2.06 y	100% 2nd line	Not reported	Mean: 47	59	60% ≥ 1 (21% 1, 24% 2, 15% ≥ 3)	Not reported
Yu 2019 [70]	Cross-sectional (single center plus patient advocacy organization, China)	Generic (TKI therapy ≥ 3 months)	1142	Dasatinib (100) Imatinib (859) Nilotinib (183)	Median 27 m	70% 1 st line 30% 2nd/3rd line	Not reported	42	63	26% (15% cardiovascular, 11% other)	Not reported

*d*, Day; *HCT-CI*, hematopoietic cell transplantation comorbidity index; *m*, month; *RCT*, randomized controlled trial; *w*, week; *y*, year

### Critical appraisal

Seven studies achieved a moderate or higher quality score, with no studies scoring the maximum score of seven because only one point was assigned to a patient-reported assessment of outcome (see Supplementary File 1). Four studies [6, 34, 41, 43] scored low on study quality because there were doubts on the representativeness of the sample, the number of non-respondents was high, or the instrument used to register symptoms was not validated.

### Meta-analyzed prevalence of patient-reported toxicity-symptoms

Eleven studies reported on 47 symptoms of any severity during imatinib, dasatinib, nilotinib, or bosutinib use, with a meta-analyzed prevalence range of 5.0 to 71.2% across symptoms (Fig. 1 in Supplementary File 1, and forest plots for all meta-analyses in Supplementary File 1). The prevalence of the symptom burden of imatinib was reported by seven different articles on 1795 patients [8, 14, 16, 22, 42, 43, 70]. Fatigue (71.2%, 95% confidence interval [95% CI]: 59.7–82.7%, *I*^2^ = 95.2, five studies), edema (69.3%, 95% CI: 60.8–77.9%, *I*^2^ = 89.5%, five studies) and muscle soreness (65.2%, 95% CI: 47.2–82.5%, *I*^2^ = 97.8, five studies) were the symptoms with the highest reported prevalence of any severity across studies. Symptoms during dasatinib use were reported by four studies [8, 22, 43, 70] with a total population of 233 patients, giving a top three symptom burden of any severity of fatigue (64.1%, 95% CI: 44.5–83.7%, *I*^2^ = 89.7, four studies), frequent urination (53.8%, 95% CI: 45.1–62.6%, *I*^2^ = 0%, two studies) and musculoskeletal pain (52.0%, 95% CI: 26.5–77.5%, *I*^2^ = 92.9%, two studies). Prevalence for nilotinib was described by six articles [6, 8, 19, 34, 43, 70] with a total population of 509 patients. Its most frequent symptoms of any severity were fatigue (67.0%, 95% CI: 59.0–75.1], *I*^2^ = 60%, five studies), frequent urination (63.3%, 95% CI: 46.1–80.1%, one study) and itchy skin (53.5%, 95% CI: 44.7–62.3%, *I*^2^ = 42.3%, two studies). Bosutinib was described by two articles [14, 41], giving a total population of 450. Only for pain and anxiety/depression of any severity, one or more studies were available, with effect estimates of respectively 37.1% (95% CI: 30.9–43.2%, *I*^2^ = 51.6%, two studies) and 44.1% (95% CI: 24.1–64.1%, *I*^2^ = 96.3%, two studies).

Heterogeneity between studies was high for most symptoms of any severity, resulting in a median heterogeneity and range of 87.3% (0.0–97.8) for imatinib, 55.3% (0.0–97.6) for dasatinib, 62.0% (0.0–90.9) for nilotinib, and 73.9% (51.6–96.3) concerning bosutinib.

Five studies reported on the proportion of 1973 patients with moderate-severe symptoms on a four point scale [16, 22, 42, 49, 70] (Fig. 2 in Supplementary File 1). The prevalence of moderate-severe symptoms during imatinib was reported by four different articles on 1475 patients [16, 22, 42, 70]. Eye problems (34.0%, 95% CI: 24.4–43.6%, one study), frequent urination (33%, 95% CI: 23.6–42.4%, one study), and edema (31.0%, 95% CI: 23.5–38.5%, *I*^2^ = 86.1%, three studies) were the three most reported moderate-severe symptoms under imatinib. Two studies reported on moderate-severe symptoms in 194 patients on dasatinib [49, 70]. Fatigue (25.3%, 95% CI: − 1.2–57.7%, *I*^2^ = 95.2%, two studies), weight change (24.0%, 95% CI: 15.6–32.4%, one study), and frequent urination (19.0%, 95% CI: 11.0–27.0%, one study) were the three most reported moderate-severe symptoms under dasatinib. The same two studies reported on symptoms during nilotinib, with the top three moderate-severe symptoms in 304 patients being fatigue (25.6%, 95% CI: 16.8–34.4%, *I*^2^ = 68.8%, two studies), itchy skin (24.0%, 95% CI: 17.7–30.3%, one study), and pain (22.0%, 95% CI: 14.6–29.5%, one study). The median heterogeneity of the studies for moderate-severe symptoms was 45.8% (0–96.4) for imatinib, 22.1% (0–95.2) for dasatinib, and 41.8% (0–91.1) for nilotinib.

### Sensitivity analysis

Excluding the four low-quality studies [6, 34, 41, 43] did not significantly alter meta-analyzed effect estimates of the most prevalent symptoms for each TKI (data not shown). Hence, no articles were left out of the meta-analysis.

### Differences in symptom prevalence between TKI types

Statistically significant more patients on imatinib experienced abdominal distension, abdominal pain, breast pain/swelling (females), a decrease in sexual desire, diarrhea, edema, dry eyes, hair color change, hypomenorrhea, muscle cramps/soreness, nausea, pain, or skin color change (any severity and/or moderate-severe), when compared to both dasatinib and nilotinib (Table 2). Musculoskeletal pain, vomiting, and weight gain were experienced more by patients on imatinib compared to nilotinib. Breast pain/swelling (females), dry eyes, hypomenorrhea, and an itchy skin were experienced more by patients using nilotinib than dasatinib. Moderate-severe memory problems were experienced more frequently by patients on dasatinib, compared to imatinib and nilotinib. More clinically relevant differences, tentatively defined as a magnitude of ≥ 10% or higher, were identified between TKI types, but none of these differences reached statistical significance of *p* < 0.01 (data not shown).

**Table 2:**
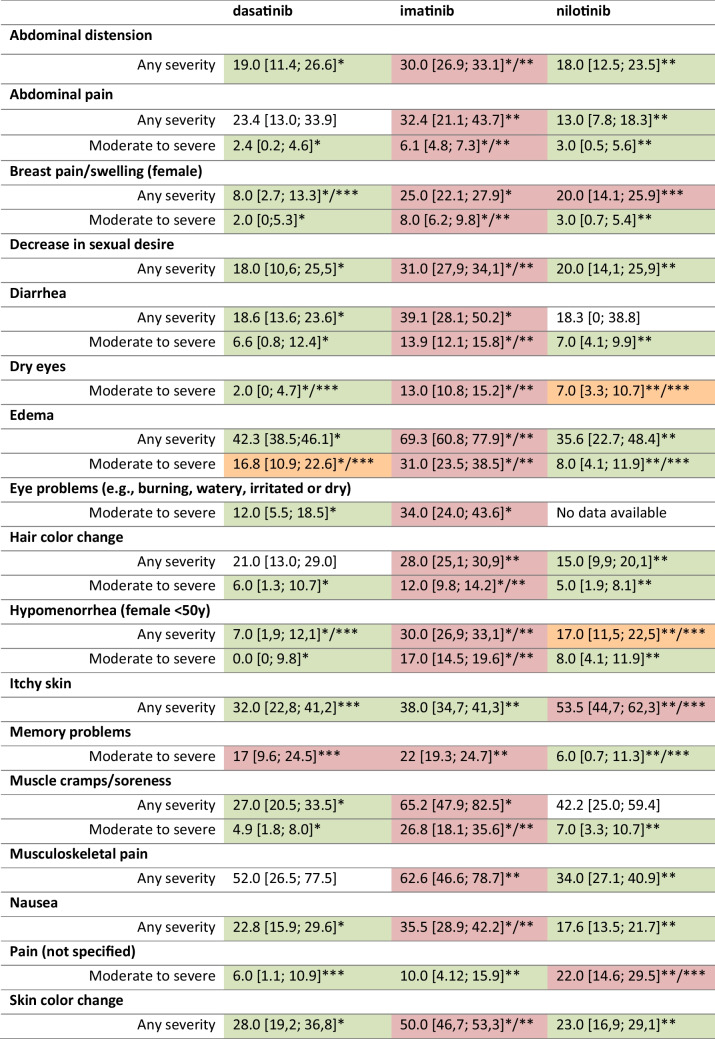

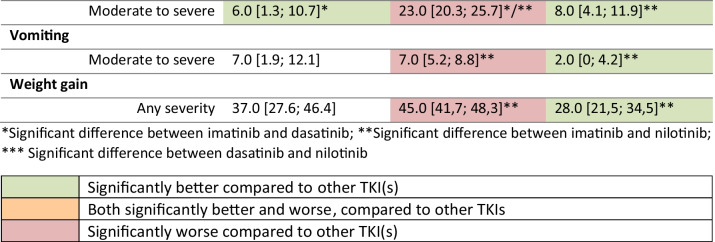
Prevalence (%) [with 95% confidence intervals] of symptoms with statistically significant differences across TKI types, based on indirect comparisons between studies

### Meta-regression

Multivariable meta-regression was not possible due to the limited number of studies that provided data on covariates. Similarly, too few data were available on comorbidity and comedication to perform meta-regression, whereas treatment duration could only be taken up in the analysis of fatigue for imatinib. Explorative meta-regression was performed on fatigue and nausea for imatinib and nilotinib and on muscle cramp, edema, and diarrhea for imatinib only, as these were the only symptoms with data on (some) covariates in at least five studies. Concerning fatigue of any severity, as reported by nilotinib users, median age seemed to explain a large part of the heterogeneity (51.8%). With a co-efficient of − 1.30, the estimated prevalence of fatigue decreased when age increased. No other significant changes were identified (data not shown).

## Discussion

This study critically investigated the symptom burden during TKI therapy, as reported by chronic phase CML patients. The prevalence of patients who experienced symptoms of any severity was reported for 47 different symptoms and ranged from 5.0 to 69.3% for different TKI types and symptoms. The key symptom was fatigue, which is in line with previous clinician-reported adverse effects [17]. In an indirect comparison of imatinib, dasatinib, and nilotinib, statistically significant differences in symptom prevalence (any severity) were identified for 13 symptoms, generally favoring dasatinib and nilotinib over imatinib. Only one included study made a direct comparison between TKI types (the BFORE randomized trial), reporting on pain and anxiety/depression of any severity only [14]. Results for these symptoms were similar to other prevalence data.

Real-world patient-reported symptoms have the potential to improve our understanding of patients’ treatment experience and, as such, provide a better picture of what influences long-term quality of life for CML patients, and could thereby improve communication between clinicians and patients [65]. Especially when switching TKI therapy for intolerance, knowledge of TKI specific patient-reported symptom burden is needed to inform shared-decision-making. Moreover, this can inform treatment choices prior to initiation. For example, if a newly treated CML patient is already suffering from diarrhea, dasatinib might be a better choice than imatinib. Of course, the patient-reported symptom burden should always be weighed against effectiveness. Furthermore, real-world data rather than clinical trials could provide information on toxicity-symptoms regarding more exclusive or rare diseases [65] and can help unravel which symptoms are truly due to TKI toxicity, and which are the (combined) effect of other patient- or medication-related factors, such as age, gender, co-medication or comorbidity. Similarly, real-world data on TKI dosage could help clarify the relationship between TKI dosage and -toxicity, but data on dosage, comorbidities, comedication, and treatment duration were not available with enough consistency (e.g., mean daily dosage) from the included studies to allow meaningful analysis. Of note, our data are not real-world data. Two of the eleven included studies were randomized controlled trials, three further studies were conducted in one or two centers only, and nine out of eleven studies described restrictive inclusion criteria such as for example: complete cytogenetic response; ECOG-performance status of 0 or 1; and/or a specific TKI as first-line therapy.

In this meta-analysis, we described a wide variety of toxicity-symptoms, which may reflect different underlying diseases that can be caused, at least in part, by TKI usage. A recent Swedish study by Dahlen et al. [15] described a variety of diseases with an increased incidence during TKI treatment, compared to controls. The disease-categories with the highest increased incidence were the circulatory, respiratory, ophthalmic, infectious, gastrointestinal, and genitourinary systems, with a specific elevated risk of cardiovascular outcomes for nilotinib and ponatinib and pleural effusion and infectious diseases for dasatinib. These results are partly reflected by the symptom burden in our meta-analysis, with, for example, eye symptoms and dyspnea being common. However, symptoms related to many of the diseases described by Dahlen et al. [15] were not, or only minimally, identified in this meta-analysis, particularly genitourinary diseases. Yu et al. [70], using a generic questionnaire, was the only study to report hyper-/hypomenorrhea. This suggests that current validated questionnaires are still not comprehensive enough to capture all symptoms.

The need to use patient-reported outcomes in CML research is emphasized when we compare the patient-reported symptom burden estimated in this study to the toxicity-burden estimated by studies that applied CTCAE, as reported by physicians (any severity). Our effect estimates are often at the upper end of or even above the range of the prevalence of all-grade toxicity assessed by CTCAE [2, 10], further supporting the idea that physician-assessed toxicity underestimates the symptoms patients’ experience. Fatigue and edema, in particular, were reported at significantly lower rates (≥ 29% (fatigue) and 16% (edema)) in CTCAE assessments for both dasatinib and nilotinib [10]. Similarly, for imatinib, muscle cramps were reported 23% less frequently [2], compared to our meta-analyzed effect estimates.

Our study is the first to quantitatively analyze the prevalence of CML patient-reported symptoms during TKI therapy. Its strength lies in the detailed analysis and variation of patient-reported symptom burden provided by combining data from different studies. In contrast to CTCAE, there is no consensus on how patient-reported outcomes should be measured and reported. Seven out of 11 identified studies used validated instruments, with only four using leukemia-specific instruments. Two of these four studies used the FACT-LEU, which is not sufficiently comprehensive, as it misses key TKI-related toxicity, such as muscle cramps [62]. However, the other validated CML specific instrument used by two studies (EORTC QLQ-CML24) does not cover genitourinary symptoms, which appear to be prevalent based on one study using a generic instrument [70]. Unfortunately, this generic instrument lacks sufficient content validity [62]. Therefore, this review does not determine which instrument is best suited to assess TKI related toxicity in CML.

As for the reporting of outcomes, we had to exclude many studies because of differences in reporting, e.g., 17 studies did not report per TKI type [4, 12, 18, 20, 24, 26, 27, 39, 44, 50, 52, 53, 60, 61, 64, 66, 72], while 19 studies reported other outcomes than prevalence [1, 3, 5, 13, 19, 29, 30, 36, 37, 46, 47, 54–57, 67, 68, 71, 73]. To some extent, we can compare our findings to 12 of these studies, which reported mean symptom scores [1, 3, 13, 19, 37, 54, 55, 57, 67, 68, 71, 73]. Eight of those 12 studies reported on multiple symptoms, identifying fatigue as the most severe symptom in dasatinib [1, 13, 73]; imatinib [1, 3, 13, 37, 54], nilotinib [1, 3, 13, 19, 57, 73]; as well as for ponatinib [73]. However, for bosutinib one study found that diarrhea had the highest mean score [3]. This suggests that fatigue is both the most prevalent and the most severe symptom, though this might not apply to bosutinib, for which we had no prevalence data.

In addition, eight of those 12 studies made comparisons between different TKI types: six observational studies made unadjusted comparisons [1, 3, 13, 67, 68, 73], while two were randomized controlled trials [55, 71]. Comparisons were made between asciminib, bosutinib, dasatinib, imatinib, nilotinib, and ponatinib. Overall, 22 different treatment groups were identified across these studies with six treatment groups including fewer than 30 patients and only two treatment groups including more than 100 patients. Findings from these eight studies were diverse: some reached statistical significance for certain comparisons, while others did not find significant differences for similar comparisons, or even reported contradictory significant differences. Notably, most studies used questionnaires that evaluated only a few separate symptoms. At present, the variety of questionnaires used and the variability in the reporting of outcomes compromise the comparability of patient-reported symptoms, making it challenging to draw consistent and clinically meaningful findings across studies [59]. Low patient numbers and unadjusted comparisons further contribute to this problem.

Two of those eight studies that compared mean severity scores but could not be included in our meta-analysis, still provide valuable supplementary data and merit discussion. The first is the randomized ASCEMBL trial, which compared asciminib to bosutinib [55]. Reporting adjusted mean differences in symptom scores using a mixed-effects model for repeated measurements, the study found that six out of 20 individual symptom items statistically favored asciminib over bosutinib (nausea, lack of appetite, feeling drowsy, dry mouth, vomiting, and diarrhea). However, the differences were small and did not reach the predefined clinical meaningful difference of 15%, except for diarrhea, which worsened under bosutinib. The second study with supplementary data found that patients on ponatinib had significantly worse mean scores compared to patients on dasatinib and/or nilotinib for skin rash, muscle cramps, dry mouth and distress, disturbed sleep, malaise, swelling of extremities, and shortness of breath [73].

The research field is at the beginning stages of leveraging real-world data, and the availability and quality of such data are still limited [31]. As a starting point, it would be useful if more and larger studies reported on toxicity-symptoms, tabulating outcomes according to TKI type and response given, instead of reporting on quality of life expressed by mean scores. Even though we identified 47 symptoms on which 11 studies reported in this way, 53% (symptoms of any severity) to 70% (moderate-severe symptoms) of all our analyses are based on the results of only one study. Furthermore, data on bosutinib, ponatinib, and asciminib were scarce or absent and are especially needed to inform clinical care.

Another limitation is the high heterogeneity of the meta-analyzed effect sizes, reflecting either clinical heterogeneity, such as population or treatment differences, and/or methodological heterogeneity, such as different questionnaires. Previous studies have shown significant correlations between patient-reported symptoms and, for example, gender, treatment duration, age, comorbidity, and comedication [16, 20, 38]. We identified age as a covariate that explained heterogeneity to a large extent for fatigue during nilotinib use. The prevalence of patient-reported fatigue of any severity decreased with increasing age, possibly due to a “response shift”, a psychological adaptation in which patients either change their internal standards for measuring a concept (what is “fatigue” may shift during the course of a chronic illness); or redefine fatigue as they age [63]. However, the limited number of studies that provided (consistent) information on pre-determined covariates restricted exploratory analysis: we did not find a similar age-related effect on fatigue prevalence during imatinib or dasatinib use, for example.

In this meta-analysis, frequent urination is found to be a high-prevalence symptom for CML patients treated by either of the three analyzed TKIs, although it has not been described as a side effect of TKIs before. Besides it possibly being an effect of TKI treatment, feasible explanations could be that it is a consequence of edema during TKI treatment, or due to comorbidity. Due to limited information on covariates, this could not be further specified. An individual-patient data meta-analysis of existing studies might shed more light on covariates. Future studies should incorporate consistent and standardized information on covariates, to help unravel the impact they have on the patient symptom-experience in the real-world.

## Conclusion

Low-grade patient-reported symptom burden during TKI usage is high, with significant differences between TKI types for a third of reported symptoms. Though evidence is mainly indirect and unadjusted for covariates, this is the most in-depth overview of patients’ experiences available to the best of our knowledge. These findings are a prerequisite for shared decision-making, when discussing treatment choices with patients. Future real-world studies should focus on direct comparisons between different TKI types, adjusted for covariates, including asciminib, bosutinib, and ponatinib.

## Supplementary Information

Below is the link to the electronic supplementary material.

ESM 1 (DOCX 583 KB)

## Data Availability

No datasets were generated or analysed during the current study.
