# Supplementary material for: Patient-reported toxicity symptoms during tyrosine kinase inhibitor treatment in chronic myeloid leukemia: a systematic review and meta-analysis
Source: Support Care Cancer. 2025 May 3;33(5):446. doi: 10.1007/s00520-025-09451-4 (PMC12049300; doi:10.1007/s00520-025-09451-4)
Supplement: Supplementary file 1 — (DOCX 583 KB) [file 520_2025_9451_MOESM1_ESM.docx]

Supplementary File 1

Patient-reported toxicity symptoms during tyrosine kinase inhibitor treatment in chronic myeloid leukemia: a systematic review and meta-analysis

*Yolba Smit^1^, *Pien Scheuter^1^, Myrthe P.M. Lange^1^, Jeroen J.W.M. Janssen^1^, Eduardus F.M. Posthuma^2^, Charlotte L. Bekker^3^, Rosella P.M.G. Hermens^4^, Nicole M.A. Blijlevens^1^

*PS and YS contributed equally to this study

**Affiliations**

^1^ Department of Hematology, Radboud university medical center, Nijmegen, the Netherlands

^2^ Department of Internal Medicine, Reinier de Graaf Hospital, Delft, Netherlands

^3^ Department of Pharmacy, Radboud university medical center, Nijmegen, the Netherlands

^4^ Department of IQ healthcare, Radboud university medical center, Nijmegen, the Netherlands

**Corresponding author**

Yolba Smit

[yolba.smit@radboudumc.nl](mailto:yolba.smit@radboudumc.nl)

# Index

[**Affiliations** 1](#_Toc191542650)

[**Corresponding author** 1](#_Toc191542651)

[1. Index 1](#_Toc191542652)

[2. Search strategy 4](#_Toc191542653)

[3. Critical appraisal 6](#_Toc191542654)

[4. Symptom definitions 6](#_Toc191542655)

[5. Figure 1 7](#_Toc191542656)

[6. Figure 2 8](#_Toc191542657)

[7. Meta-analyses: forest plots per symptom (any severity) 9](#_Toc191542658)

[1. Fatigue 9](#_Toc191542659)

[2. Edema 10](#_Toc191542660)

[3. Muscle soreness and cramps 11](#_Toc191542661)

[4. Eye problems 12](#_Toc191542662)

[5. Musculoskeletal pain 13](#_Toc191542663)

[6. Frequent urination 14](#_Toc191542664)

[7. Acid indigestion 15](#_Toc191542665)

[8. Skin color change 16](#_Toc191542666)

[9. Weight change 17](#_Toc191542667)

[10. Dry mouth 18](#_Toc191542668)

[11. Weight gain 19](#_Toc191542669)

[12. Pain 20](#_Toc191542670)

[13. Diarrhea 21](#_Toc191542671)

[14. Dyspnea 22](#_Toc191542672)

[15. Itchy skin 23](#_Toc191542673)

[16. Drowsiness 24](#_Toc191542674)

[17. Insomnia 25](#_Toc191542675)

[18. Hidrosis 26](#_Toc191542676)

[19. Dry eyes 27](#_Toc191542677)

[20. Dizziness 28](#_Toc191542678)

[21. Memory problems 29](#_Toc191542679)

[22. Anxiety and depression 30](#_Toc191542680)

[23. Rash and/or skin problems 31](#_Toc191542681)

[24. Nausea 32](#_Toc191542682)

[25. Palpitations 33](#_Toc191542683)

[26. Headache 34](#_Toc191542684)

[27. Hair loss 35](#_Toc191542685)

[28. Abdominal pain 36](#_Toc191542686)

[29. Malaise 37](#_Toc191542687)

[30. Tinnitus 38](#_Toc191542688)

[31. Decrease in sexual desire 39](#_Toc191542689)

[32. Hypomenorrhea (female <50y) 40](#_Toc191542690)

[33. Abdominal distension 41](#_Toc191542691)

[34. Distress 42](#_Toc191542692)

[35. Numbness/tingling 43](#_Toc191542693)

[36. Hair color change 44](#_Toc191542694)

[37. Bruising and/or bleeding easily 45](#_Toc191542695)

[38. Breast distending pain (females) 46](#_Toc191542696)

[39. Sadness 47](#_Toc191542697)

[40. Depression 48](#_Toc191542698)

[41. Appetite loss 49](#_Toc191542699)

[42. Vomiting 50](#_Toc191542700)

[43. Hypermenorrhea (females<50y) 51](#_Toc191542701)

[44. Weight loss 52](#_Toc191542702)

[45. Constipation 53](#_Toc191542703)

[46. Gynecomastia (males) 54](#_Toc191542704)

[47. Amenorrhea (females<50y) 55](#_Toc191542705)

# Search strategy

The search strategy included terms and synonyms for ‘chronic myeloid leukemia’, ‘protein kinase inhibitors’ and different TKI brand names. Case reports, editorials and phase I clinical trials were excluded in the search string and the language of the articles was restricted to English. Phase I clinical trials were excluded as these are dose finding studies: they usually do not evaluate patient reported symptoms, and when they do, they would only involve small numbers of patients over a very short period of time.

|  | **Search string** | **January 2022** | **July 2023** | **February 2025** |
| --- | --- | --- | --- | --- |
| **Pubmed** | | **Citations** |  |  |
| #1 | "Leukemia, Myelogenous, Chronic, BCR-ABL Positive"[MeSH] OR “chronic myelogenous leukemia” OR “chronic myeloid leukemia” OR “Ph1 positive chronic myelogenous” OR “Ph1-positive chronic myelogenous” OR “Ph1 positive chronic myelogenous” OR “Ph1-positive chronic myelogenous” OR “Philadelphia positive chronic myeloid leukemia” OR “Philadelphia-positive chronic myeloid leukemia" OR “Chronic myelocytic leukemia” OR “CML” | 35,356 | 37,107 | 38,951 |
| #2 | "Protein Kinase Inhibitors"[MeSH] OR “protein kinase inhibitor” OR “protein kinase inhibitors” OR “TKI” OR “TKIs” OR “TKI’s” OR “imatinib” OR "imatinib mesylate"[MeSH] OR “Glivec” OR “nilotinib” OR “Tasigna” OR “dasatinib” OR “sprycel” OR “bosutinib” OR “bosulif” OR “ponatinib” OR “iclusig” OR “asciminib” | 134,109 | 150,783 | 95,356 |
| #3 | #1 AND #2 | 10,173 | 10,966 | 11,659 |
| #4 | "case reports"[Publication Type] OR "clinical trial, phase i"[Publication Type] OR "editorial"[Publication Type] |  |  |  |
| #5 | #3 NOT #4 | 8,299 | 8,954 | 9,521 |
| #6 | humans[Filter] AND english[Filter] |  |  |  |
| #7 | #5 AND #6 | **6,562** | 7,104 | 7,471 |
| #8 | Filters: from 2021 - 2023(search July 2023 only) |  | **806** |  |
| #9 | Filters: from 2023 – 2025 (search February 2025 only) |  |  | **630** |
| **EMBASE OVID** | |  |  |  |
|  |  |  |  |  |
| 1 | chronic myeloid leukemia.mp. or exp chronic myeloid leukemia/ | 50,577 | 54,279 | 57,897 |
| 2 | cml.mp. | 31,136 | 33,384 | 35,486 |
| 3 | 1 or 2 | 58,604 | 62,806 | 66,930 |
| 4 | tyrosine kinase inhibitor.mp. or exp protein tyrosine kinase inhibitor/ | 340,117 | 411,877 | 466,369 |
| 5 | tki.mp. | 23,153 | 27,470 | 31,374 |
| 6 | (imatinib or glivec).mp. or exp imatinib/ | 47,035 | 50,929 | 54,170 |
| 7 | (nilotinib or tasigna).mp. or exp nilotinib/ | 10,425 | 11,733 | 13,041 |
| 8 | (dasatinib or sprycel).mp. or exp dasatinib/ | 15,937 | 18,282 | 20,673 |
| 9 | (bosutinib or bosulif).mp. or exp bosutinib/ | 3,070 | 3,697 | 4,272 |
| 10 | (ponatinib or iclusig).mp. or exp ponatinib/ | 3,611 | 4,519 | 5,455 |
| 11 | asciminib.mp. or exp asciminib/ [mp=title, abstract, heading word, drug trade name, original title, device manufacturer, drug manufacturer, device trade name, keyword heading word, floating subheading word, candidate term word] | 204 | 422 | 748 |
| 12 | 4 or 5 or 6 or 7 or 8 or 9 or 10 or 11 | 343,570 | 415,833 | 470,885 |
| 13 | 3 and 12 | 24,939 | 27,145 | 29,177 |
| 14 | exp note/ or editorial/ | 1,510,164 | 1,637,632 | 1,743,631 |
| 15 | 13 not 14 | 23,692 | 25,827 | 27,785 |
| 16 | limit 15 to (human and english language and exclude medline journals) | **2,507** |  |  |
| 16 | Limit 15 to (human and English language and “ remove medline records” and yr=”2021 -Current” (July 2023 search only) |  | **1,342** |  |
| 16 | Limit 15 to (human and English language and “remove medline records” and yr=”2023 -Current” (Feb 2025 search only) |  |  | **1,001** |
|  |  |  |  |  |
|  | **Pubmed and Embase combined** | **9,069** | **2,148** | **1,631** |
|  | Identified through reference tracking | 1 | 0 | 1 |
|  | Subtotal | 9,070 | 2,148 | 1,632 |
|  | De-duplication between Pubmed and Embase records | 8,988 | 2,039 | 1,632 |
|  | Duplicates between January 2022 and July 2023 searches removed (July 2023 search only) |  | 1,465 |  |
|  | Duplicates between July 2023 and Feb 2025 searches (Feb 2025 search only) |  |  | 1,302 |
|  | Total from both searches |  | |  |
|  | **Screened on title and abstract** | **8,988** | **1,465** | **1,302** |
|  | Excluded on title and/abstract | 8,566 | 1,449 | 1,263 |
|  |  |  |  |  |
|  | **Screened full text** | **422** | **16** | **39** |
|  | Excluded after full text screening: | 412 | 15 | 39 |
|  | *No data on specific symptoms* | *21* | *1* | *0* |
|  | *No (original) patient reported data* | *365* | *10* | *25* |
|  | *No data per TKI* | *11* | *2* | *4* |
|  | *No prevalence data* | *13* | *1* | *5* |
|  | *Patient reported symptoms after/during TKI withdrawal* | *1* | *1* | *4* |
|  | *Only severe symptoms reported* | *1* | *0* | *0* |
|  | *Only data on top 3 symptoms per patient* | *0* | *0* | *1* |
|  |  |  |  |  |
|  | **Included after full text screening:** | **10** | **1** | **0** |
|  | *Asciminib* | *0* | *0* | *0* |
|  | *Bosutinib* | *2* | *0* | *0* |
|  | *Dasatinib* | *4* | *0* | *0* |
|  | *Imatinib* | *7* | *0* | *0* |
|  | *Nilotinib* | *5* | *1* | *0* |
|  | *Ponatinib* | *0* | *0* | *0* |

# Critical appraisal

Table 1 Critical appraisal of included studies, according to adapted Newcastle Ottawa Scale

| Study | Selection | | | | Comparability | Outcomes | | Total |
| --- | --- | --- | --- | --- | --- | --- | --- | --- |
|  | **Representati-veness sample**  **(max 1 star)** | **Sample size**  **(max 1 star)** | **Non-respondents**  **(max 1 star)** | **Ascertainment of exposure**  **(max 2 stars)** | **Control confounding factors** | **Assessment of outcome**  **(max 2 stars)** | **Statistical test** | **0-7 stars** |
| Boons  2020 [24] |  | * | * | * | n.a. | * | n.a. | 4 |
| Bostan  2020 [17] |  | * | * | ** | n.a. | * | n.a. | 5 |
| Cortes  2019 [18] | * | * | * | ** | n.a. | * | n.a. | 6 |
| Efficace  2011 [19] | * | * | * | ** | n.a. | * | n.a. | 6 |
| Efficace 2020 [20] | * | * | * | ** | n.a. | * | n.a. | 6 |
| Huguet 2019 [26] |  | * |  | ** | n.a. | * | n.a. | 4 |
| Kantarjian  2018 [27] |  | * |  | ** | n.a. | * | n.a. | 4 |
| Kapoor  2015 [23] |  | * | * | ** | n.a. | * | n.a. | 5 |
| Kekale  2015 [21] |  | * | * | * | n.a. | * | n.a. | 4 |
| Nguyen  2022[28] | * | * |  | ** | n.a. | * | n.a. | 5 |
| Yu  2019 [22] | * | * | * | * | n.a. | * | n.a. | 5 |

Abbreviation: n.a.: not applicable

# Symptom definitions

In so far as symptom definitions are not obvious, included terms are described here.

| General term | Included terms |
| --- | --- |
| Bruising/bleeding easily | Bruising easily; easy bruising/bleeding; hemorrhagic tendency of skin |
| Edema | Swelling of ankles, legs or around eyes; swelling of hands, legs, feet, or around eyes; periorbital and lower limb edema; edema |
| Rash and/or skin problems | Rash/skin changes; rash; skin problems |
| Pain | Pain; pain and discomfort |

# Figure 1

Figure 1 Meta-analyzed effect estimates (weighted average) of the prevalence of 47 patient reported symptoms of any severity, reported by 2,987 patients across 11 studies on bosutinib, dasatinib, imatinib, and nilotinib

*Significant difference between dasatinib and imatinib; **Significant difference between imatinib and nilotinib; *** Significant difference between dasatinib and nilotinib

# Figure 2

Figure 2 Meta-analyzed effect estimates (weighted average) of the prevalence of 43 patient reported symptoms of moderate to severe intensity, reported by 1,973 patients across five studies on dasatinib, imatinib and nilotinib

*Significant difference between dasatinib and imatinib; **Significant difference between imatinib and nilotinib; *** Significant difference between dasatinib and nilotinib

# Meta-analyses: forest plots per symptom (any severity)

### Fatigue

### Edema

### Muscle soreness and cramps

### Eye problems

### Musculoskeletal pain

### Frequent urination

### Acid indigestion

### Skin color change

### Weight change

### Dry mouth

### Weight gain

### Pain

### Diarrhea

### Dyspnea

### Itchy skin

### Drowsiness

### Insomnia

### Hidrosis

### Dry eyes

### Dizziness

### Memory problems

### Anxiety and depression

### Rash and/or skin problems

### Nausea

### Palpitations

### Headache

### Hair loss

### Abdominal pain

### Malaise

### Tinnitus

### Decrease in sexual desire

### Hypomenorrhea (female <50y)

### Abdominal distension

### Distress

### Numbness/tingling

### Hair color change

### Bruising and/or bleeding easily

### Breast distending pain (females)

### Sadness

### Depression

### Appetite loss

### Vomiting

### Hypermenorrhea (females<50y)

### Weight loss

### Constipation

### Gynecomastia (males)

### Amenorrhea (females<50y)
